# Level of physical activity among middle-aged and older Chinese people: evidence from the China health and retirement longitudinal study

**DOI:** 10.1186/s12889-020-09671-9

**Published:** 2020-11-10

**Authors:** Xiaowei Li, Wanda Zhang, Weiya Zhang, Ke Tao, Wenli Ni, Kai Wang, Zhanglai Li, Qiang Liu, Jianhao Lin

**Affiliations:** 1 Peking University People’s Hospital, No. 11 Xizhimen South Street, Xicheng District, Beijing, 100044 CN China; 2 Division of Rheumatology, Orthopaedics and Dermatology, University of Nottingham, Nottingham, UK; 3 Arthritis Pain Centre, University of Nottingham, Nottingham, UK; 4 School of Public Health, Peking University Health, Beijing, CN China

**Keywords:** Public health, Physical inactivity, China

## Abstract

**Background:**

With data from different regions accumulated, physical inactivity (PI) was found to be pandemic worldwide. Using China Health and Retirement Longitudinal Study (CHARLS), a nationwide longitudinal survey data, we aimed to delineate the prevalence, incidence and risk factors of physical inactivity (PI) among Chinese people aged 45 years and older.

**Methods:**

The CHARLS covered nearly all provinces, autonomous regions, municipalities of mainland China. With data from CHARLS, three cross-sectional analyses and a cohort analysis were conducted. In cross-sectional studies, we used surveys at 2011, 2013 and 2015 to examine the prevalence and its trend of PI. Multivariate generalized linear model was conducted in survey at 2011 to examine the risk factors for prevalent PI. Multiple imputation of missing values was used and results before and after imputation were compared. In cohort analysis, we identified people free of PI at 2011 and followed them up until 2015 to estimate the incidence of PI. Generalized estimating equation was used to examine the risk factors associated with incidence PI. In all analyses, PI was defined as insufficient physical activity according to the International Physical Activity Questionnaire (IPAQ) criterion.

**Results:**

6650, 5946 and 9389 participants were eligible for cross-sectional analyses, and 4525 participants were included for cohort analysis. The weighted prevalence of PI was 22.25% (95% CI: 20.63–23.95%) in 2011, 20.64% (95% CI: 19.22–22.14%) in 2013 and 19.31% (95% CI: 18.28–20.38%) in 2015. In multivariate analysis, PI was associated with older age, higher education, overweight, obesity and difficulties in daily living, and was negatively associated with working and higher level of expenditure. No material change was detected in results after multiple imputation. In cohort analysis, older age, abundant public facilities, difficulties in daily living were identified as risk factors of incidence PI, while urban areas, college and above education, and working were protective factors.

**Conclusions:**

PI is pandemic in 45 years and older people in China. People with older age, difficulties in daily living and people who are not working are at higher risk. More efforts should be paid in estimating and promoting leisure-time physical activities.

## Background

Since the industrial revolution, people’s life styles have changed greatly throughout the world, with the amount of physical activity (PA) decreasing significantly and sedentariness becoming a major part of daily living [1]. The relief of heavy PA came at a severe cost in terms of increasing burden of chronic non-communicable diseases. Regarding the importance of PA, a series of recommendation were made to highlight the least amount of activity necessary to maintain health, which in return introduced a definition of “insufficient physically active” (IPA), also known as “physical inactivity” (PI) (the term “PI” or “physical inactivity” used in this article is exactly the same meaning as “insufficient physically active”) [2, 3]. With data from different regions accumulated, PI was found to be pandemic worldwide, affecting 31% of people globally, and correlates were successively identified, such as older age, female sex, and living in high income countries [4]. In further studies, PI was found to account for more than 5 million deaths worldwide in 2008 [5] and was identified as the fourth leading risk factor for non-communicable diseases in 2009 [6]. According to present evidence, the prevalence of PI is still increasing, and efficient interventions are needed to control this trend [1, 4].

China has nearly one fifth of the world’s population. Considering the rapid aging of the population in recent years [7], it can be inferred that Chinese people are at high risk of developing PI. Yet the data of PA in China was insufficient, and most studies were limited by representativeness [8–11]. Although data from WHO reported lower prevalence of PI in China, compared with those highly developed countries, more surveys are still necessary, not only to reveal the trend of prevalence, but also to search for risk factors of PI among Chinese that might help making interventions. A nationwide survey is now available to help address some of these limitations regarding the assessment of physical activity in China. The China Health and Retirement Longitudinal Study (CHARLS) covered nearly all provinces, autonomous regions and municipalities of mainland China (except for Tibet) and got as many as 17,705 participants involved in the baseline survey by face-to-face interview and standardised questionnaire [12].

In this study, we aim to estimate the prevalence of PI and associated risk factors among Chinese people aged 45 years and older using data from CHARLS [12].

## Materials and methods

In this study, both cross-sectional and longitudinal analyses were conducted.

### Participants and public involvement

The baseline survey of CHARLS was conducted between June 2011 and March 2012. A multistage probability sampling strategy and probability-proportional-to-size (PPS) sampling technique were employed to ensure national representativeness. Sampling was conducted at four levels. First, 150 county-level units were randomly selected using the PPS technique from a sampling frame containing all county-level units (excluding those from Tibet) and stratified by region, urban/rural status, and gross domestic product (GDP). Second, 3 primary sampling units (PSUs), defined as the lowest level of government organization, were randomly selected within each chosen county-level unit via the PPS technique. Third, 24 households were randomly selected within each PSU using mapping and listing operations. Finally, if the selected household had at least one person 45 years of age or older, then one of the persons, along with his or her spouse (if any), was randomly selected. Data on PA (only a randomly selected subsample answered questions about PA) and other characteristics (health, economy, working and retirement, etc.) were then collected using a face-to-face computer-assisted personal interview (CAPI) and physical measurements. Two follow-up surveys were conducted in 2013 and 2015. In each follow-up survey, the original participants were revisited, and new participants were selected using the same methods mentioned above to make up for people who dropped out and ensure representativeness. Detailed information about the purpose, design, sample and the questionnaires of the CHARLS is available in other articles [12].

**Fig. 1:**
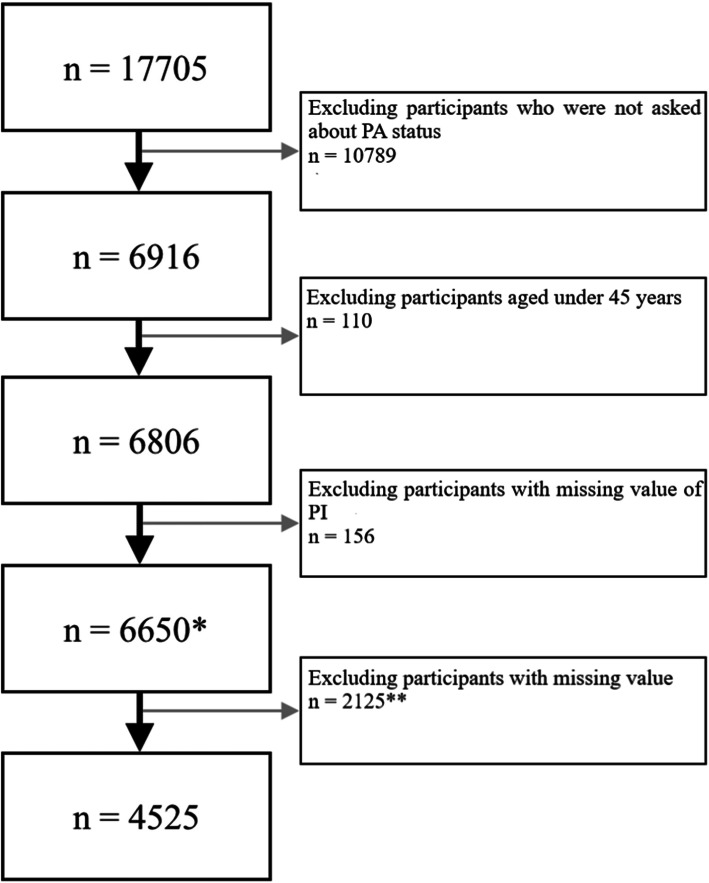
Participants selected from baseline survey in 2011. *: These participants were involved to calculate the prevalence of PI, but were not eligible for further analyses. **: Some participants have more than 1 variable with missing value

Only part of the participants was randomly selected to answer questions about PA and were thus involved in the present study. According to CHARLS, people aged less than 45 years were still possible to be involved, as long as their spouses were selected. However, all participants under the age of 45 years were excluded from data analysis in this study. Besides, all participants with one or more missing values in any variables were also excluded. Detail procedure of selection is shown in Fig. 1.

### Ethics approval

The original CHARLS was approved by the Ethical Review Committee of Peking University in June 2008, and all participants signed informed consent at the time of participation.

### Measurements

The included participants were inquired about the intensity (vigorous, moderate and walking), time of duration (more than 4 h, 2–4 h, 30 min-2 h, and less than 30 min), and days (1–7) they did physical activities in a usual week. The questionnaire used in this study is part of the questionnaire of CHARLS, which can be found in the CHARLS questionnaire on page 70–71.The structure and statement of this section of questionnaire were similar to the short version of International Physical Activity Questionnaire (IPAQ), a globally recognized questionnaire designed to measure total PA across all domains [13] There were three differences between CHARLS and IPAQ: First, PA information of “a usual week” instead of “the last 7 days” were collected. Previous study showed similar performance of the two reference periods [13]. Second, no information on sedentariness were collected. Third, four discrete time durations instead of continuous value were collected. To implement calculation, the time duration of each intensity level was replaced by the median of each group (the group “more than 4 hours” was replaced by 4 h), and the amount of different intensity levels were then summed using the metabolic equivalent (MET) as a reference, the weight of each intensity was derived from IPAQ scoring protocol [14]. Only activities lasting for more than 10 min at one time were counted [12]. Both questionnaires were listed and compared in the appendix. The equations are listed below. Although the IPAQ was originally designed for people up to aged 69 years, its validity in older people was proved adequate and it was believed to be a useful tool for assessing physical activity among elderly adults [15].

Walking MET-minutes/week = 3.3*walking minutes*walking days

Moderate MET-minutes/week = 4.0*moderate-intensity activity minutes*moderate days

Vigorous MET-minutes/week = 8.0*vigorous-intensity activity minutes*vigorous-intensity days

Total PA MET-minutes/week = sum of Walking + Moderate + Vigorous MET-minutes/week scores [14].

PI was identified if PA amount in a week failed to meet at least one criterion of the “moderate” level according to IPAQ group, described as follows:

a) 3 or more days of vigorous-intensity activity of at least 20 min per day

OR

b) 5 or more days of moderate-intensity activity and/or walking of at least 30 min per day

OR

c) 5 or more days of any combination of walking, moderate-intensity or vigorous intensity activities achieving a minimum total PA of at least 600 MET-minutes/week [14].

As potential risk factors, data regarding demographic characteristics (age, gender, marital status, living area), socioeconomic status (SES) (educational level, annual per capita expenditure [PCE], working status), health-related habits (smoking status, alcohol intake), health status (non-communicable diseases, difficulties of daily living), physical environment (public facilities), and body mass index (BMI) were also obtained and further stratified or dichotomized. The corresponding subcategories are listed in Table 1. Marital status was dichotomized as having a spouse or not. Quintiles of PCE were used to assess economic status by dividing participants into 5 groups. Apart from non-smokers, smokers were further classified according to whether they had quit smoking at the time of the interview. Alcohol intake status was divided into 3 groups: never drinking alcoholic beverages, drinking less than once a month and drinking more than once a month. Subjects were asked whether they had ever been diagnosed with the following diseases: hypertension, dyslipidaemia, diabetes or high blood sugar, cancer or malignant tumour, chronic lung disease, liver disease, heart problems, stroke, kidney disease, stomach or other digestive disease, emotional, neural or psychiatric disease, memory-related disease, arthritis or rheumatism, and asthma. Difficulties in daily living was measured by Activities of Daily Living (ADL) and Instrumental Activities of Daily Living (IADL) questions, which were two common measures used for estimating disability among elderly people [16]. Participants who reported of having any degree of difficulties in performing at least one activity listed in the ADL and IADL questions were identified as “ADL/IADL disability”. Same definition has been reported in other studies [16, 17]. According to the suggested categories for Asian populations, BMI values were classified into “underweight” (< 18.5 kg/m^2^), “normal weight” (18.5–23), “overweight” (23–27.5), and “obese” (≥27.5) [18].

**Table 1:** Characteristics of the baseline sample and the distribution of PI

Variables	Number of total participants	Number of physically inactive participants	Prevalence of PI	*χ*^2^	*p* value
Age				91.474	< 0.001
45–59	2323	371	16.0%		
60–74	1821	357	20.0%		
≥ 75	381	140	36.8%		
Gender				8.264	< 0.001
Male	2085	362	17.4%		
Female	2440	506	20.7%		
Spouse				8.968	< 0.001
Yes	3820	704	18.4%		
No	705	164	23.3%		
Living area				25.910	< 0.001
Urban	1648	381	23.1%		
Rural	2877	487	16.9%		
BMI level				25.227	< 0.001
Underweight	300	72	24.0%		
Normal	1888	308	16.3%		
Overweight	1720	338	19.7%		
Obese	617	150	24.3%		
Work status				332.581	< 0.001
Currently working	3179	389	12.2%		
Not working	1346	479	35.6%		
Education level				4.165	0.24
Illiteracy	1242	256	20.6%		
Elementary school	1846	330	17.9%		
Middle/high school	1360	265	19.5%		
College or above	77	17	22.1%		
PCE level				4.750	0.31
1/5	935	200	21.4%		
2/5	940	171	18.2%		
3/5	904	163	18.0%		
4/5	923	182	19.7%		
5/5	823	152	18.5%		
Smoking				7.114	0.03
Never	2796	562	20.1%		
Quit	368	77	20.9%		
Yes	1361	229	16.8%		
Drinking				13.888	< 0.001
Never	3040	629	20.7%		
< Once a month	339	58	17.1%		
> Once a month	1146	181	15.8%		
Non-communicable diseases^a^				0.487	0.49
Yes	3083	600	19.5%		
No	1442	268	18.6%		
ADL/IADL disability				49.277	< 0.001
Yes	1241	321	25.9%		
No	3284	547	16.7%		
Number of public facilities	3.44 ± 3.39	/	/	/	/

Categorical data was presented by frequency (n%), quantitative data was presented by mean value and standard deviation. Weighting was conducted using individual level weights with non-response adjustment released by the CHARLS. *χ*^2^: results of Chi-square tests. ^a^Participants who have been diagnosed of having at least one of the diseases listed in Measurement section were categorized into “yes”

### Statistical analyses

The prevalence of PI was calculated in baseline survey and 2013, 2015 follow-up surveys. To better reflect the target population, we repeated the calculation and used individual weights with adjustment of non-response to adjust the sampling probability, and reported both results as the prevalence of PI. The weights, provided by CHARLS, were created through sampling probability with an inverse probability weighting factor constructed to correct for nonresponse and sampling-frame errors [12].

In the baseline survey, differences of prevalence of PI between sub-populations were analysed with a Pearson Chi-square test (for homoscedasticity) or Fisher’s exact test (for heteroscedasticity) for categorical variables and Analysis of Variance (ANOVA) (for homoscedasticity) or the Kruskal-Wallis test (for heteroscedasticity) for continuous variables. Besides, when analysing the relationship between PI and socioeconomic status, to eliminate the confounding factor of working status, we stratified participants by their working status. In each stratum, PI’s prevalence in each level of education and expenditure was calculated and compared with the lowest level (i.e., reference level) using a one-factor binary logistic regression model. In addition, cross-strata differences were evaluated by Chi-square test within each education/PCE level.

To determine the correlates of PI, a multi-variable analysis was conducted. Empirically, PI is pandemic, thus the odds ratio may overestimate the relative risk [19]. Therefore, we used a Generalized linear model with Poisson distribution, log link and robust variance estimate to implement analysis. In the model, the presence of PI was set as dependent variable and the other as independent variables. Because a large proportion of participants were excluded due to missing data, we performed multiple imputation by chained equations (MICE) and repeated the main analyses [20]. We assumed data was missed at random, and results before and after multiple imputation were compared.

**Fig. 2:**
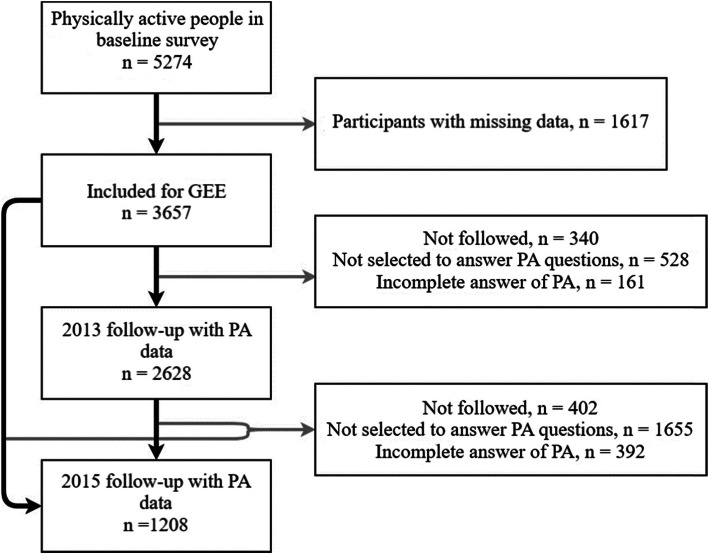
Participants selected for the cohort analysis

Additionally, a cohort analysis was conducted to evaluate the incidence and risk factors of PI. Participants who was physically active in the baseline survey (*n* = 3657) were followed up in 2013 and 2015for incidence PI. Because being physical active/inactive is a temporary status, which can shift over time, we define “occurrence of PI” as physically active people in 2011 survey being detected PI in either 2013 or 2015 survey. The incidence density of PI was calculated, and participants who dropped out in either 2013 or 2015 survey, no matter what the reason was, were excluded in the calculation. A generalized estimating equation (GEE) was employed to examine the temporal association between baseline exposure factors, and new cases of PI identified in either 2013 or 2015 (Fig. 2.)

All tests were two-sided, and in all analysis’s procedures, a significant difference was defined as *p* < 0.05. Procedures were performed using SPSS ver.24 and Stata ver.13.

## Results

In total 6650, 5946 and 9389 participants were included in cross-sectional analyses in surveys at 2011, 2013 and 2015, respectively to calculate the prevalence of PI. The average age was 60.2 ± 9.7 in 2011, 60.5 ± 9.8 in 2013, and 60.5 ± 10.0 in 2015. In survey at 2011, after excluding participants with missing values in potential risk factor variables, a total of 4525 eligible participants were included in multivariate analysis. The characteristics of the baseline sample (*n* = 4525) are shown in Table 1. The average age was 60.0 ± 9.4 years, the oldest participant was 93 years old. Of these, 3657 were at risk (i.e., had no PI) were included in the cohort analysis.

**Fig. 3:**
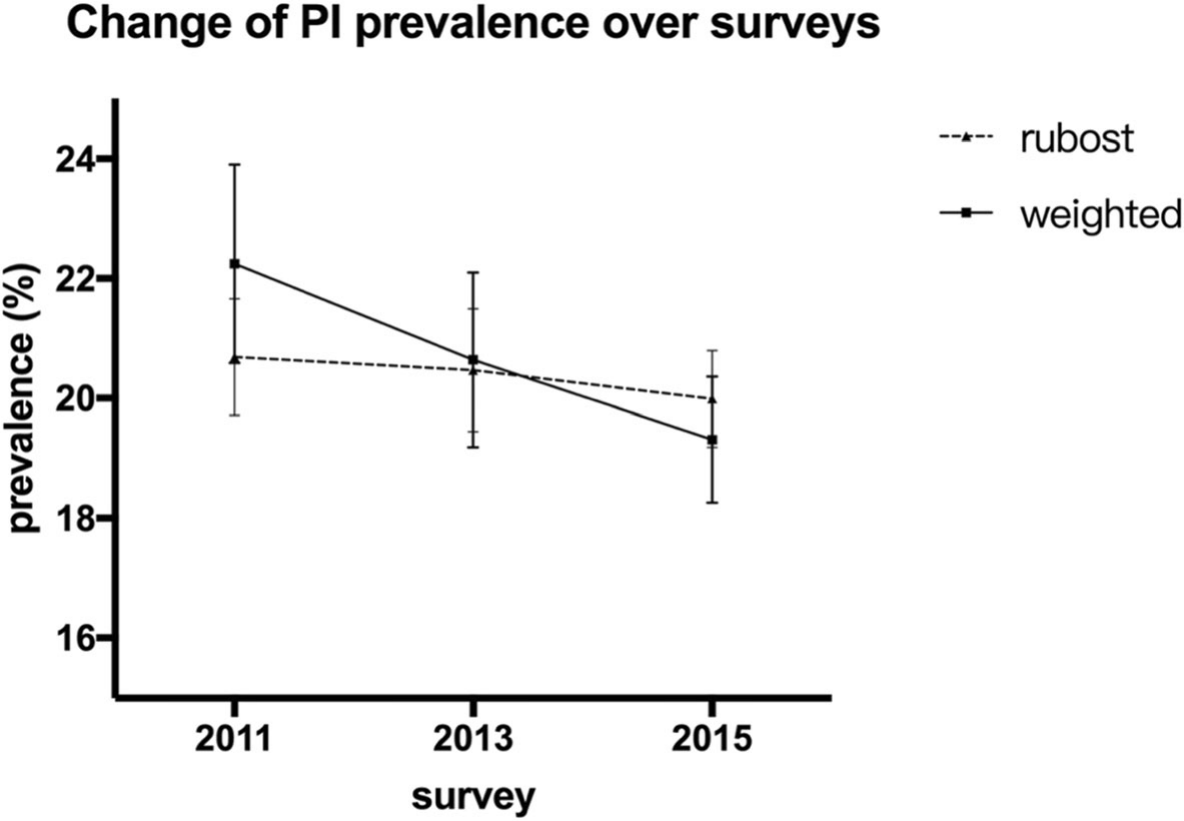
Change of PI prevalence over surveys

**Fig. 4:**
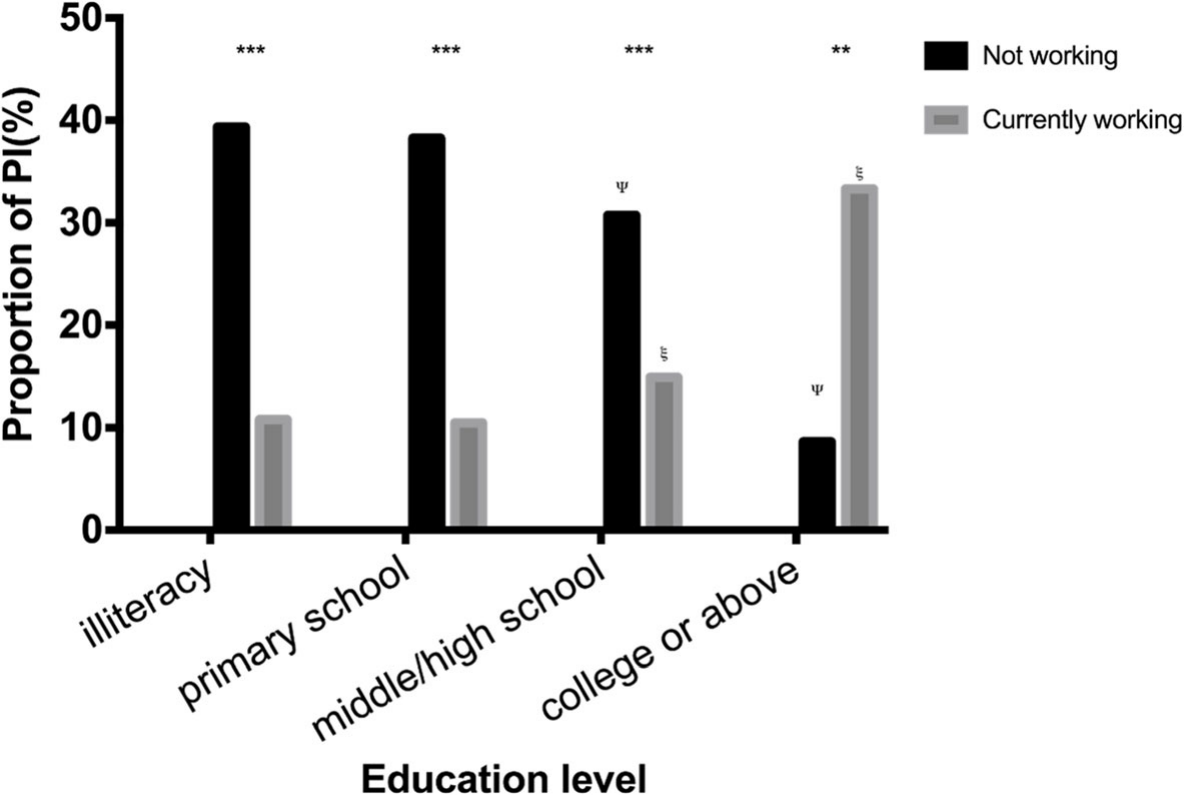
Proportion of PI in the groups stratified by working status and education levels

**Fig. 5:**
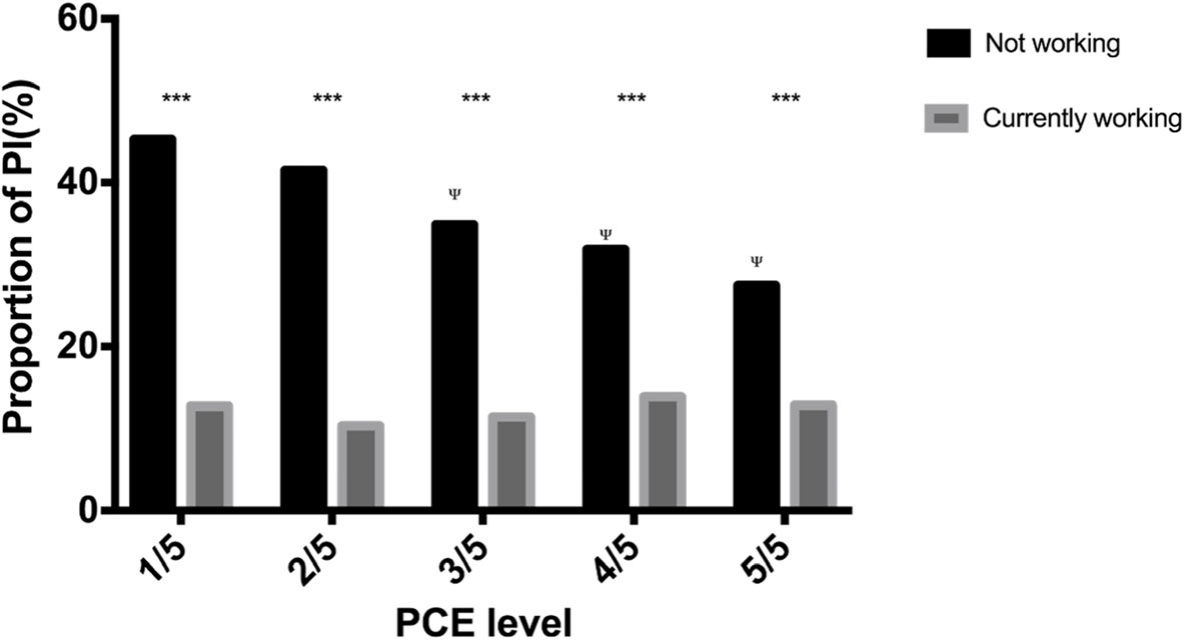
Proportion of PI in the groups stratified by working status and PCE levels

As shown in Fig. 3, the prevalence of PI showed no significant difference between the three time points of data collection, with a *p* value of 0.530 (chi2: 1.271). The detailed distribution of the prevalence of PI in baseline survey and its relationship with demographic characteristics are shown in Table 1. The highest prevalence of PI was among people ≥75 years (36.75%) and people who were not working (35.59%). PI correlates with most of those characteristics except for education level and PCE level. Yet, when participants were stratified by working status, the trend of PI prevalence with education levels or expenditure levels were opposite, as shown in Figs. 4 and 5: Among people who were working (grey columns), the proportion of PI increased with higher educational level (Fig. 4) and remained with higher PCE level (Fig. 5); By contrast, among people who were not working (black columns), the proportion of PI decreased with higher educational level (Fig. 4) or higher PCE level (Fig. 5).

Results of multivariate analysis of baseline survey before and after multiple imputation are shown in Table 2. A large scale of observations of PCE levels (*n* = 945) and BMI levels (*n* = 1161) were missed, but imputation of missing data did not materially change the estimated associations. Working status was one of the major correlations of PI, working people were about 60% less likely to be insufficiently active than people who were not working.

**Table 2:** Risk factors associated with prevalent PI

	Missing observations	Robust		MI	
		*p* value	RR (95% CI)	*p* value	RR (95% CI)
PI	156	/	/	/	/
Age	0				
45–60		Ref	Ref	Ref	Ref
60–75		0.82	0.98 (0.86 to 1.13)	0.801	0.99 (0.88 to 1.10)
> =75		< 0.001	1.35 (1.11 to 1.64)	< 0.001	1.38 (1.20 to 1.60)
Gender	2				
Male		Ref	Ref	Ref	Ref
Female		0.47	1.06 (0.90 to 1.25)	0.869	1.01 (0.89 to 1.15)
Living area	0				
Rural		Ref	Ref	Ref	Ref
Urban		0.16	1.10 (0.96 to 1.26)	0.091	1.10 (0.99 to 1.23)
Spouse	0				
No		Ref	Ref	Ref	Ref
Yes		0.93	0.99 (0.85 to 1.16)	0.607	0.97 (0.87 to 1.09)
Education level	3				
Illiteracy		Ref	Ref	Ref	Ref
Elementary school		0.36	1.07 (0.92 to 1.24)	0.850	1.01 (0.90 to 1.13)
Middle/high school		0.03	1.22 (1.02 to 1.45)	0.188	1.10 (0.96 to 1.26)
College or above		0.51	1.18 (0.72 to 1.93)	0.787	1.05 (0.74 to 1.50)
Working status	21				
Not working		Ref	Ref	Ref	Ref
Currently working		< 0.001	0.38 (0.33 to 0.44)	< 0.001	0.43 (0.38 to 0.48)
PCE level	945				
1/5		Ref	Ref	Ref	Ref
2/5		0.12	0.87 (0.73 to 1.04)	0.154	0.90 (0.77 to 1.04)
3/5		0.04	0.83 (0.70 to 0.99)	0.057	0.86 (0.74 to 1.00)
4/5		0.11	0.87 (0.73 to 1.03)	0.040	0.86 (0.74 to 0.99)
5/5		0.01	0.77 (0.63 to 0.93)	0.003	0.78 (0.66 to 0.92)
Public facilities	119	0.28	0.99 (0.97 to 1.01)	0.033	0.98 (0.97 to 1.00)
BMI level	1161				
Normal		Ref	Ref	Ref	Ref
Underweight		0.05	1.25 (1.00 to 1.55)	0.003	1.33 (1.10 to 1.60)
Overweight		0.02	1.18 (1.03 to 1.35)	0.008	1.19 (1.05 to 1.34)
Obesity		< 0.001	1.31 (1.11 to 1.56)	0.003	1.27 (1.09 to 1.48)
NCDs	272				
No		Ref	Ref	Ref	Ref
Yes		0.05	0.88 (0.78 to 1.00)	0.057	0.90 (0.81 to 1.00)
Smoking	0				
Never smoking		Ref	Ref	Ref	Ref
Used to smoke, have stopped		0.82	0.97 (0.78 to 1.22)	0.277	0.91 (0.76 to 1.08)
Currently smoking		0.92	1.01 (0.85 to 1.19)	0.661	0.97 (0.85 to 1.11)
Drinking	1				
Never drinking		Ref	Ref	Ref	Ref
Less than once a month		0.60	0.94 (0.74 to 1.19)	0.510	0.94 (0.78 to 1.13)
More than once a month		0.85	0.98 (0.83 to 1.16)	0.129	0.90 (0.78 to 1.03)
ADL/IADL disability	94				
No		Ref	Ref	Ref	Ref
Yes		< 0.001	1.36 (1.20 to 1.54)	< 0.001	1.44 (1.31 to 1.59)

The analysis was conducted using cross-sectional data of baseline survey. *MI* Multiple imputation, *RR* relative risk, *CI* Confidence interval, *ref* Reference group, *PCE* Per capita expenditure, *PA* physical activity, *BMI* Body mass interval, *NCD* non-communicable diseases

The overall incidence density of PI was 7.59% per person-year from 2011 to 2015. Further, Table 3 summarises the results of the GEE, whereby advanced age, abundant facilities in the community, and difficulties in daily living were positively associated of developing PI, and people who lived in urban areas, who were working and who had received college or higher education were not likely to develop PI.

**Table 3:** Risk factors associated with the incident PI

Variables	RR(95%CI)	*p* value
Age (years)		
45–60	Ref	Ref
60–75	1.23 (1.03 to 1.46)	0.02
≥ 75	1.65 (1.21 to 2.26)	< 0.001
Female	0.92 (0.73 to 1.14)	0.44
Urban	0.73 (0.6. to 0.90)	< 0.001
Having a spouse	0.82 (0.67 to 1.01)	0.06
Education levels		
Illiteracy	Ref	Ref
Elementary school	0.92 (0.76 to 1.11)	0.37
Middle/high school	0.83 (0.65 to 1.06)	0.13
College or above	0.39 (0.16 to 0.93)	0.03
Currently working	0.67 (0.55 to 0.83)	< 0.001
PCE		
1/5	Ref	Ref
2/5	0.91 (0.72 to 1.15)	0.43
3/5	1.01 (0.80 to 1.28)	0.94
4/5	0.84 (0.65 to 1.07)	0.15
5/5	0.84 (0.64 to 1.09)	0.19
Public facilities	1.03 (1.01 to 1.07)	0.02
BMI levels		
Underweight	1.08 (0.78 to 1.48)	0.65
Normal	Ref	Ref
Overweight	1.15 (0.96 to 1.37)	0.13
Obese	1.24 (0.97 to 1.58)	0.09
NCDs	0.99 (0.83 to 1.18)	0.93
Smoking status		
Never	Ref	Ref
Quit	0.95 (0.69 to 1.31)	0.75
Yes	1.00 (0.8 to 1.24)	0.98
Alcohol		
Never	Ref	Ref
< Once a month	1.00 (0.74 to 1.36)	1.00
> Once in a month	0.85 (0.69 to 1.06)	0.14
ADL/IADL disabilities	1.22 (1.03 to 1.46)	0.03

*RR* relative risk, *CI* confidence interval, *ref* reference group, *PCE* per capita expenditure, *PA* physical activity, *BMI* body mass index, *NCD* non-communicable diseases

## Discussion

This study contains a cross-sectional analysis and a cohort analysis, in order to examine the prevalence and incidence of PI in China. The main findings are [1] one in five people aged over 45 had PI in China [2]; the prevalence remained stable between 2011 to 2015 [3]; the main risk factors associated with the prevalent PI were ≥ 75 years old, not working, higher expenditure, abnormal BMI, having disabilities in daily living [4]; incidence density of PI was 7.59 per 100 person years [5]; the main risk factors for incident PI were older age, abundant facilities, disabilities in daily living, rural area, not working, and not receiving college or above education.

The prevalence of PI reported in this article is similar to the estimate for Chinese women (18.4%) from the 2010 CCDRFS, another nation-wide dataset in China [11]. Though, these two studies both used nationwide datasets, the participants’ characteristics were quite different. The study based on CCDRFS investigated only women with a minimum age of 18 years old, while the present study included males and females over the age of 45 years. The results of these two studies draw us a rough picture about the prevalence of PI in China. According to this prevalence, over 80 million people in China have PI [7]. However, compared to other countries, especially highly developed countries, PI is far less pandemic in China. In a review by Hallal [4] using a similar criterion with this study, nearly one in three people (31.1%) in the world were found inactive, and the prevalence ranged from 33.7% in the Western Pacific region, to 34.8% in Europe, 43.2% in the Eastern Mediterranean region and 43.3% in America. The actual difference could be more significant because the participants in Hallal’s review [4] were 15 years and older instead of 45 years and older in our study. Such a difference is consistent with previous studies [21–23], which might be partly explained by the increased participation in manual labour. Data acquired from World Bank show a higher employment rate in China (67.9%) than in Britain (56.8%) and Northern America (57.8%) among people aged 15+ years [24]. According to the baseline report of the CHARLS, at least 20% of rural people over the age of 80 years are still working [25].

Previous reported prevalence of PI in China was difficult to compare due to different criteria of PI and different sampling strategies [8–11]. After searching on the Internet, two studies were found using similar criteria with our study. The first study used data from the World Health Survey (WHS) whereby in a sample of Chinese adults (mean age 42.2 years), 9.3% of males and 11.8% of females were inactive [21]. The other study used representative data from Shanghai, whereby participants aged from 18 to 65 years old had an overall PI prevalence of 6.9% [23]. Compared to these two studies, the prevalence of PI reported in the current study was approximately twice as high. The rates of PI have in essence doubled in the past decade (both previous studies were conducted between 2002 and 2004), or the older mean age result in the high prevalence of PI is a possible reason. The mean amount of PA of Chinese people was also found decreasing in the past decades, from 388 MET hours/week in 1991 to 213 MET hours/week in 2009 [26]. However, findings in the present study suggests that PI prevalence was decreasing, from 22.25% in 2011 to 19.31% in 2015. There is a possible interpretation for the contradiction: Due to relatively short period of follow-up, we might have mistaken little fluctuate in the prevalence of PI for the real trend. According to data from the population census of China, people aged 65 years and over accounted for 7.37% of the population in 2000, and the proportion has increased to 8.92% in 2010 [7]. A number of studies have reported the rapid urbanization of China in recent years, accompanied by a decline in the employment rate, changes in working patterns from manual labour such as agriculture and handicraft to sedentary work such as office work, and the appearance and spread of labour-saving techniques [26–29]. All of these changes may have led to a substantial decrease in occupational PA (OPA). It is believed that urbanization has accounted for 57% of the decrease in PA among males and 40% among females, and in the OPA domain, the proportion is higher, at approximately 4/5 among males and 2/3 among females [30].

In general, the distribution pattern of PI is consistent with previous studies in China or other countries [4, 21, 31, 32]. But in cross-sectional analyses, no association was found between PI and education level or PCE level, which was believed to be negatively correlated with PI in several studies [8, 29, 33]. However, when people were stratified according to working status, PI was found negatively associated with education level and PCE level among people who were not working while the association was positive among people who were still working. The most reasonable interpretation might be the heavy burden of work or the long time occupied by working of Chinese people. According to previous studies in this field, OPA composed the major part of total PA in Chinese people [26, 28, 29]. Empirically, higher SES levels typically signify non-manual jobs, which are less physically demanding [27]. In fact, people with higher incomes were observed to have lower OPA [29], which eventually leads to lower total PA. On the other hand, people who were not working do not have OPA and are thus expected to have a higher proportion of leisure-time physical activity (LTPA), our findings are in accordance with previous studies that LTPA is positively correlated with PI [8, 33]. A significant decrease in PI only appeared for middle/high school or higher levels of education and 3/5 or higher levels of PCE, highlighting the risk of PI among people in the lowest SES sublevels who should not be ignored by the government.

A significant and positive association was detected between working status and PI, suggesting that non-working people were three times more likely to be inactive than working people; this finding further strengthens the opinion that OPA is the main contributor to total PA in developing countries [4, 28, 29]. It seems that people fail to find an adequate substitute for OPA after exiting from work. OPA has been shown to promote health and be a partial source of energy expenditure [34, 35], but according to present evidence, OPA has been decreasing rapidly during last 2 decades. As the urbanization process continues, the trend will likely be maintained, which may result in loss of a substantial amount of overall PA [26, 28, 29].. In developed countries, LTPA in adults is increasing, whereas OPA seems decreasing [35, 36] In China, a slight growing trend of LTPA was detected, but the amount was not comparable with OPA [26]. Apart from making up for the reduction of OPA [37], LTPA is also believed to have unique benefits on health, including decreasing the risk of cancer and lowering all-cause death [38, 39]. Thus, the trend of decreasing OPA may be unavoidable; instead, stimulating LTPA in China should be priority, not only to maintain the amount of overall PA, but also to change the pattern of PA to become more health-promoting.

Using panel data from the CHARLS from 2011 to 2015, we used a GEE to identify the determinants of PI. Most of the results parallel findings of cross-sectional analyses and previous studies, suggesting that people with older age, no job and difficulties in daily living are more likely to develop PI [4, 21, 31, 32]. Additionally, living in urban areas was found to be protective against developing PI. This finding may be partly due to better access to PA facilities in more urbanized areas [29, 40, 41]. People with college or above education levels were 61% less likely to develop PI than illiterate people, which was consistent with previous studies [8, 35, 42]. Self-efficacy, more access to facilities, and better ability to utilize available sources might be reasons for this finding [27, 29, 42, 43]. Surprisingly, we found that each additional facility in a community/village resulted in an approximately 3% higher risk of developing PI. Although difficult to believe in empirical view, this result is similar to that of other studies conducted in China [29, 30] and is actually not contradictory to other existing studies that focused solely on LTPA and identified a positive association with an improved environment [44, 45]. One hypothesis is that more abundant public facilities reflects a more favourable physical environment, but not all environmental attributes are positively related to PA [40]. For instance, highly developed public transportation and labour-saving technologies may decrease PA associated with commuting and OPA [4]. From this point of view, our findings may suggest an insufficient LTPA-promoting effect of the environment in China. Another reason might be the heterogeneity of the involved facilities in this study. Both PA-promoting facilities such as basketball courts and Ping-Pong rooms and PA-reducing facilities such as chess rooms were counted. According to previous studies, promoting walkability, including building sidewalks, improving the proximity of amenities and the aesthetic environment, and providing sufficient lighting could possibly stimulate walking and subsequently increase the amount of PA. These factors should be considered by the government when investigating environment-related projects [40, 46, 47].

This study has several strengths. First, because the results are derived from a nationally representative dataset with a large sample size, they can largely reflect the actual status of PI in middle-aged and older Chinese people. Second, with follow-up data, continuous change of the prevalence of PI from 2011 to 2015 was revealed. Third, by longitudinal analyses, we are able to evaluate the causal relationship between PI and potential risk factors, which is more convincing than a cross-sectional analysis.

### Study limitations

First, with present questionnaires of the CHARLS, we cannot acquire domain-specific data of PA for separate analyses. However, we dichotomized the sample according to work status and therefore excluded the confounding impact of OPA in people who were not working. The distinct distribution of PI in people with/without jobs suggests that the correlates of LTPA and OPA might be quite different in China, like in other regions [4, 32, 37, 42]. Second, while the IPAQ has been a very commonly used tool to measure physical activity, some recent studies demonstrate that the IPAQ overestimates levels of PA compared with objective physical activity measuring devices including accelerometers [4, 48]. This may mean that the levels of physical activity/inactivity reported in this study may not be completely accurate. However, the use of the IPAQ still allows a comparison of the data in this study to other large-scale Chinese studies that used this tool. Further, use of self-reported questionnaires such as the IPAQ remain the only feasible way of collecting such data from this number of people over several years [4]. Third, data about PA derived from the CHARLS were self-reported, which entails some error in recall. One factor that mitigates this concern is that previous studies have validated the reliability of self-reported PA [4, 13, 21–23]. Finally, approximately one-third of participants (2281 of 6806) were excluded because of missing data, which might have caused some selection bias. However, we repeated cross-sectional analyses after multiple imputation of missing data, and the results were largely similar to the original results, indicating that data were missed randomly and did not affect conclusion.

## Conclusion

Our findings suggest that PI has become a serious public health problem in China, affecting about one in five Chinese individuals aged 45 years and older, which deserves great attention and active intervention. Working status is the most significant correlate and determinant of PI, which adds to the fact that OPA is still the major part of PA. However, as urbanization continues, the proportion of OPA will probably continue to decrease. Strategies should specifically focus on promoting LTPA to compensate for the decrease in OPA and to gain unique health benefits. Among Chinese individuals aged 45 or older, older age, having difficulties in daily living, and not working are high-risk factors of PI. Further studies with domain-specific data are needed.

## Data Availability

Technical appendix, statistical code and dataset available from the CHARLS repository, http://charls.pku.edu.cn.

## Abbreviations

ADL: Activities of Daily Living
CHARLS: China Health and Retirement Longitudinal Study
IADL: Instrumental Activities of Daily Living
IPAQ: International Physical Activity Questionnaire
MET: metabolic equivalent
PA: physical activity
PI: physical inactivity
